# What is mental wellbeing?

**DOI:** 10.1192/bjo.2021.631

**Published:** 2021-06-18

**Authors:** David Baldwin, Julia Sinclair, Gemma Simons

**Affiliations:** 1 Professor of Psychiatry and Head of Mental Health Group, University of Southampton Faculty of Medicine Honorary Professor of Psychiatry, University of Cape Town, South Africa Visiting Professor, Shandong Mental Health Centre, China; 2 Centre for Workforce Wellbeing, University of southampton

## Abstract

**Aims:**

To explore the theory of wellbeing and to propose an operational definition for wellbeing in doctors.

Hypothesis: An operational definition for wellbeing in doctors is needed in order for it to be measured and interventions to improve it developed.

**Background:**

There is no internationally recognised definition for wellbeing and yet wellbeing is an increasingly fashionable topic of research and development, including in doctors. This is because wellbeing can be described using either hedonist, or eudonist philosophy and there is a lack of conceptual clarity about what wellbeing is, and how it works. Research into the measurement of mental wellbeing has been dominated by individualist societies, with the inherent bias towards measuring self-centred components and not the other-orientated components that might be valued more in collectivist societies and by doctors.

**Method:**

The Centre for Workforce Wellbeing (C4WW), a collaboration between the University of Southampton and Health Education England, was created to support research into the nature, assessment and enhancement of wellbeing in physicians. A literature review of the philosophy, definition and measurement of wellbeing was undertaken with a focus on mental wellbeing at work and specifically in doctors.

**Result:**

A concept map of the relationship between wellbeing terms has been created and was used to understand and classify where mental wellbeing itself was being defined and measured in studies, as opposed to a component of wellbeing, or determinant of wellbeing. Thematic analysis was used to develop an operational definition of wellbeing for doctors.

**Conclusion:**

Measurement of wellbeing and interventions for wellbeing cannot be developed if you cannot clearly define what wellbeing is. An operational definition of mental wellbeing in doctors is ethically required to prevent research waste and to allow us to identify and recreate when doctors thrive, not just survive.

Health Education England funded PhD.

